# ABC Transporters in T Cell-Mediated Physiological and Pathological Immune Responses

**DOI:** 10.3390/ijms22179186

**Published:** 2021-08-25

**Authors:** Christoph Thurm, Burkhart Schraven, Sascha Kahlfuss

**Affiliations:** 1 Institute of Molecular and Clinical Immunology, Medical Faculty, Otto-von-Guericke University Magdeburg, 39120 Magdeburg, Germany; christoph.thurm@med.ovgu.de (C.T.); burkhart.schraven@med.ovgu.de (B.S.); 2 Health Campus Immunology, Infectiology and Inflammation (GCI-3), Medical Faculty, Otto-von-Guericke University Magdeburg, 39120 Magdeburg, Germany; 3 Institute of Medical Microbiology and Hospital Hygiene, Medical Faculty, Otto-von-Guericke University Magdeburg, 39120 Magdeburg, Germany

**Keywords:** ABC transporters, T cells, Th1 cells, Th2 cells, Th17 cells, Immunometabolism, metabolism, immunity to infection, autoimmunity, allergy, bacteria, fungi

## Abstract

ATP-binding cassette (ABC) transporters represent a heterogeneous group of ATP-dependent transport proteins, which facilitate the import and/or export of various substrates, including lipids, sugars, amino acids and peptides, ions, and drugs. ABC transporters are involved in a variety of physiological processes in different human tissues. More recent studies have demonstrated that ABC transporters also regulate the development and function of different T cell populations, such as thymocytes, Natural Killer T cells, CD8^+^ T cells, and CD4^+^ T helper cells, including regulatory T cells. Here, we review the current knowledge on ABC transporters in these T cell populations by summarizing how ABC transporters regulate the function of the individual cell types and how this affects the immunity to viruses and tumors, and the course of autoimmune diseases. Furthermore, we provide a perspective on how a better understanding of the function of ABC transporters in T cells might provide promising novel avenues for the therapy of autoimmunity and to improve immunity to infection and cancer.

## 1. Introduction: The ABC Transporter Family

**Figure 1:**
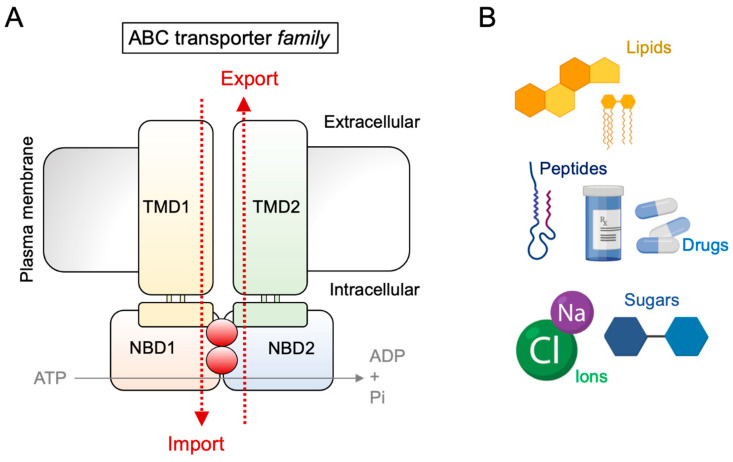
ATP-binding cassette (ABC) transporters represent a heterogeneous group of ATP-dependent transport proteins. (**A**) The illustration shows the structure of ABC transporters. (**B**) The cartoon provides an overview of typical ABC transporter substrates.

A prerequisite for the function of every cell is the directed transport of molecules and ions across lipid membranes independent of their physicochemical properties. This transport requires energy and specialized proteins when membrane permeability for specific substrates is missing or when the transport is directed against a concentration gradient. One of the largest gene families of such transport proteins are ATP-binding cassette (ABC) transporters. Human ABC transporters share common structural features. They possess two nucleotide-binding domains (NBDs), responsible for the binding of ATP, and two transmembrane domains (TMDs) (Figure 1A). In humans, ABC transporters exist either as full transporters, in which all four domains are located within the same polypeptide chain, or they form complexes of two half transporters that contain one NBD and one TMD [1]. To facilitate the transport of distinct substrates, ATP needs to be bound and hydrolyzed at NDBs of ABC transporters. The energy gained from this process is needed to induce conformational changes within the TMDs, which ultimately allows substrates to cross the lipid bilayer [2].

ABC transporter expression has been reported for several human tissues and other organisms such as bacteria, fungi, parasites, and plants. In the latter, ABC transporters were shown to control processes such as multidrug resistance, capsule synthesis, growth, and development [3,4,5,6]. In humans, 49 ABC transporter subtypes have been identified so far, which were categorized into seven subfamilies based on their amino acid sequence, protein structure, and phylogenetic origin (ABCA, ABCB, ABCC, ABCD, ABCE, ABCF, and ABCG).

ABC transporters can transport various substrates, including sugars, ions, amino acids, complex peptides, and hydrophobic (lipophilic) molecules, including lipids [7,8] (Figure 1B). Within the immune system, Transporter associated with Antigen Processing (TAP)1 and TAP2, which belong to the subclass B of ABC transporters (ABCB2 and ABCB3, respectively), are crucial for antigen processing and loading onto major histocompatibility complex I (MHC I) molecules [9]. Furthermore, outside of the immune system, the generation of bile acid in the liver is dependent on several different ABC transporters, including ABCB4 [10,11]. Besides physiological processes, ABC transporters are also involved in a number of pathological conditions. For example, ABCC7, also known as CFTR (Cystic Fibrosis Transmembrane Conductance Regulator), is an ABC transporter expressed in airway epithelial cells that conducts chloride (Cl^−^) and bicarbonate (HCO^3−^). Mutations in ABCC7 cause the development of cystic fibrosis, in which the ion equilibrium is disturbed, leading to respiratory failure due to recurrent infections [12]. Additionally, ABC transporters, such as ABCB1, ABCG2, and ABCC1, are upregulated in many cancer types [13,14].

## 2. ABC Transporters in the Development of T Cell Subsets (T Cells, Natural Killer T Cells, Tregs)

**Figure 2:**
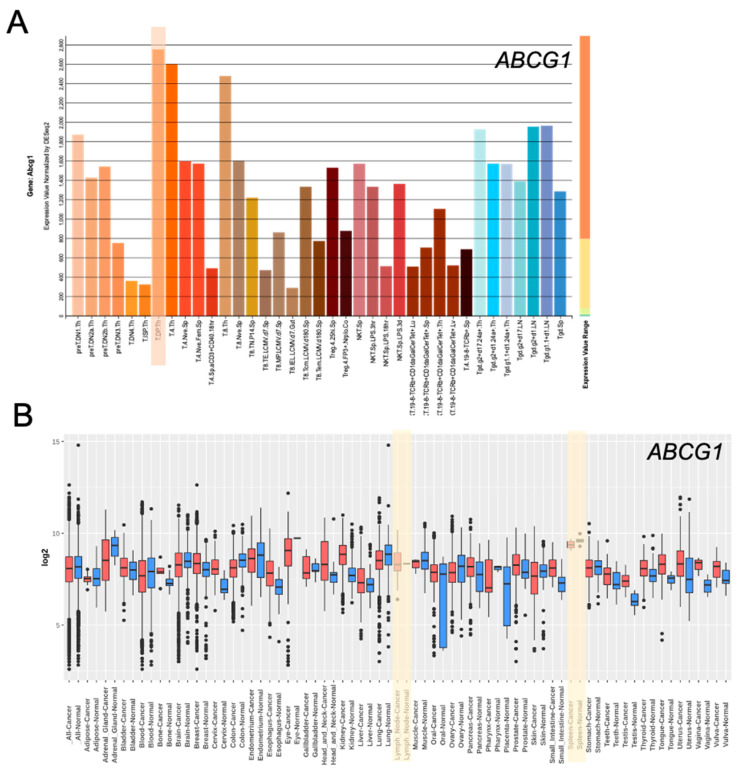
ABCG1 expression is high in double positive (DP) thymocytes and within the spleen. (**A**) Abcg1 expression for different T cell subpopulations shown as Expression Value normalized by DESeq2 and analyzed by using the ImmGen database and online platform (http://rstats.immgen.org/Skyline/skyline.html, accessed on 7 July 2021). (**B**) ABCG1 expression in different tissues analyzed by using the GENT2 platform to explore gene expression in normal and tumor tissues (http://gent2.appex.kr/gent2/, accessed on 7 July 2021).

Immune responses need to be tightly regulated to ensure immunity to pathogens but in parallel to prevent detrimental immune reactions directed against foreign (allergy) or self (autoimmunity) antigens. T cells are crucial immune cells involved in orchestrating the induction, execution, and termination of adaptive immune responses. The generation of naïve CD4^+^ and CD8^+^ T cells takes place in the thymus. During this process, thymocytes are selected based on the ability of their individual T cell receptors (TCRs) to transmit appropriate signals. The intensity and strength of TCR signals is strongly dependent on the composition and structure of the plasma membrane [15,16,17]. Changes in the lipid composition of the plasma membrane, thus, directly affect TCR-mediated signaling and, consequently, also thymocyte development. Some ABC transporters specifically transport lipids. For example, ABCG1 has a specificity for cholesterol, which is of major importance for TCR signaling [15,18]. ABCG1 was shown to be involved in thymic development of T cells. Genetic deletion of ABCG1 altered thymocyte development by increasing the proliferation of thymocytes at the transition from the double negative (DN) to the double positive (DP) stage, which cumulated in increased numbers of CD4^+^ single positive (SP) thymocytes. In line with this observation, *Abcg1* shows highest expression at the DP stage when compared to other thymocyte subpopulations in mice (Figure 2A). However, peripheral ABCG1^−/−^ CD4^+^ T cells were also reported to exhibit enhanced expansion [19]. Especially within the spleen, mRNA expression of *ABCG1* is elevated compared to other tissues (Figure 2B). Mechanistically, due to cholesterol accumulation the lack of ABCG1 expression improved proximal and distal TCR signaling, reflected by elevated phosphorylation of the downstream signaling molecules Zap70 and Erk1/2 [19]. Conversely, it was shown that anti-inflammatory liver X receptor (LXR) signaling reduces T cell proliferation via upregulation of ABCG1 [20]. However, compared to conventional T cells, ABCG1 was shown to have opposing effects on proliferation during NKT cell (Natural Killer T cell) development. Here, the absence of ABCG1 cumulated in reduced NKT cell numbers because of impaired maturation and proliferation. ABCG1-deficient NKT cells further had a decreased content of lipid rafts and showed reduced IL-4 but enhanced IFN-γ production [21]. In addition to ABCG1, loss of ABCA7 was demonstrated to impair NKT cell activation and development due to modulation of CD1d expression on DP thymocytes and thymic dendritic cells [22].

## 3. ABC Transporters in CD8^+^ T Cells and CD8^+^ T Cell-Mediated Immunity

CD8^+^ T cells mediate immunity to viruses and tumors. Viral infections lead to the generation of a small pool of memory CD8^+^ T cells, which quantitatively and qualitatively improves the immune response upon reencounter of the same virus [23,24,25,26,27]. In this regard, ABCB1, an ABC transporter known for its role in mediating multidrug resistance [28], was shown to regulate CD8^+^ T cell memory function [29]. The authors conducted a systematic screen of ABCB1 expression in various cell types and found increased expression of ABCB1 especially in CD8^+^ effector memory and central memory T cells. In their study, deletion of ABCB1 impaired acute CD8^+^ T cell activation and expansion and cumulated in an inability to generate memory CD8^+^ T cell pools. Mechanistically, ABCB1 was found to be important for mitochondrial fitness by protecting against oxidative stress during early activation of CD8^+^ T cells and memory formation [29]. Another study found that ABCB1 and ABCG2 were important for the homeostasis and function of tissue-resident memory T (Trm) cells [30], a subset of memory T cells responsible for immune surveillance in non-lymphoid tissues. Deficiency of ABCB1 and ABCG2 increased the number of CD8^+^ and CD4^+^ Trm cells in Peyer’s patches and was accompanied by increased IFN-γ production of these cells [30].

## 4. ABC Transporters in CD4^+^ T Helper Cell Populations

**Figure 3:**
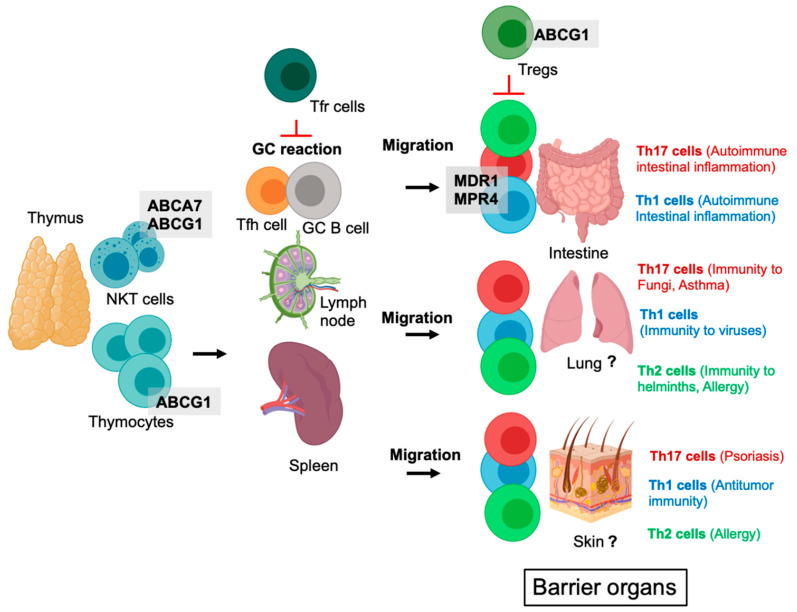
ABC transporters regulate the function of different T cell populations. Abbreviations: NKT, Natural Killer T cell; Tfh cells, T follicular helper cells; Tfr cells, T follicular regulatory cells; GC, Germinal center; Tregs, regulatory T cells; Th cell, T helper cell. MDR1, Multidrug-resistance-protein-1; MPR4, Multidrug-resistance-associated-protein-4.

Pathogenic T helper (Th)17 cells are involved in several autoimmune diseases, including MS and Crohn’s disease [31], while physiological Th17 cell immune responses mediate immunity to fungi such as *Candida albicans* [32] and extracellular bacteria [33]. Pathogenic Th17 cells were shown to be dependent on the ABC transporters ABCB1 (also known as multidrug resistance protein 1, MDR1) and the multidrug-resistance-associated-protein-4 (MRP4) [34] (Figure 3). In their study, the authors found that upon hypoxia, the transcription factor hypoxia-inducible-factor-1alpha (HIF-1α) induced MDR1 and MRP4 expression, which promoted the efflux of aryl-hydrocarbon-receptor (AhR) ligands such as unconjugated bilirubin (UCB) in Th17-cells [34]. As AhR ligands negatively regulate the pathogenic potential of Th17 cells in Crohn’s disease [35,36], increased export of UCB through MDR1 and MRP4 could, vice versa, amplify Th17 cell autoimmune intestinal inflammation during hypoxia. In line with this hypothesis, pharmacological blockade of MDR/MRP4 enhanced the immunoregulatory effect of UCB in dextran-sulfate-sodium-induced experimental colitis [34]. MDR1 was, furthermore, shown to impair oxidative stress and to control homeostasis of effector Th1 and Th17 cells in the ileum when exposed to conjugated bile acids (CBAs) [37] (Figure 3). MDR1-deficient effector T cells when transferred into Rag1^−/−^ recipient mice induced a Crohn’s-like ileitis, which could be attenuated by blocking ileal reabsorption of CBAs [37].

Furthermore, the ABC transporter MRP1 was demonstrated to be selectively upregulated in Th1 cells upon activation, while Th2 cells showed a constitutive expression of this ABC transporter [38]. In this study, pharmacological inhibition of MRP1 also suppressed T cell activation. However, this pharmacological inhibition of T cell activation was likely not a consequence of the blockade of MRP1, but of another off-target, as the authors later found that T cells from MRP1^−/−^ mice did not have a defect in T cell activation or Th1 and Th2 differentiation. Moreover, MRP1^−/−^ mice were equally susceptible to Th1 *Leishmania major* infection as WT controls [39].

## 5. ABC Transporters in Tregs

Physiological immune responses require a constant equilibrium between activation and inhibition. During acute infection, T cell activation must outbalance inhibition, whereas in the absence of pathogenic stimuli, T cell inhibition must prevail. Tregs are central cellular regulators within the immune system, which can suppress innate and adaptive immune cells. Recent observations suggest that cholesterol homeostasis in T cells and its regulation via ABCG1 directly contributes to the development of Tregs [40]. Knockout of ABCG1 in all T cells, but specifically in Tregs, was found to lead to a selective expansion of Tregs, while other T cell subsets remain unaffected [40] (Figure 3). In this study, ABCG1 was detected to be important for cholesterol cycling in Tregs as ABCG1 deficiency evoked an increase in cellular cholesterol. In turn, elevated cholesterol inhibited mammalian Target of Rapamycin (mTOR) signaling and favored Signal transducer and activator of transcription (STAT)5 signaling, which cumulated in enhanced Treg development in the absence of ABCG1. Consecutively, accelerated generation of Tregs due to ABCG1 deficiency protected from chronic inflammation in atherosclerosis [40]. In line with these observations, an inverse correlation between ABCG1 expression and the number of Tregs was also observed in humans [40]. Furthermore, TCR activation and subsequent gene expression was demonstrated to be negatively correlated with the export of cholesterol from activated T cells [19]. This was attributed to decreased expression of ABCG1 and ABCA1 following T cell activation and has been linked to the activation of the protein-tyrosine kinases Lck and Zap70 as well as the activation of the mitogen-activated protein (MAP) kinases Extracellular-signal Regulated Kinases (ERK)1/2 [19,41,42,43].

## 6. ABC Transporters in Immune Cells during Cancer

The protection against tumor development and the elimination of tumor cells is another crucial task of the immune system, which involves not only T cells but also innate immune cells, such as macrophages [44,45]. Of note, to suppress the function of immune cells, tumors have developed strategies to reprogram the anti-tumor machinery [46,47]. ABCA1 was identified as a tumor suppressor, as it favors the execution of cell death programs via mitochondrial pathways and limits AKT signaling by regulating the lipid composition in the plasma membrane [48]. In line with this, missense mutations in *ABCA1* conferring a proliferative advantage have been identified in patients with chronic myelomonocytic leukemia [49]. On the other hand, cancer cells can modulate ABCA1 expression in tumor infiltrating immune cells. As shown by Goossens and colleagues using a mouse model for high-grade serous ovarian cancer, tumor-associated macrophages are reprogrammed by hyaluronic acid towards an anti-inflammatory M2 phenotype. This is achieved by altering cholesterol metabolism and homeostasis, likely via the modulation of ABCA1 activity [50]. Interestingly, the cholesterol metabolite 27-Hydroxycholesterol (27-HC), which is produced by various tumors, has also been shown to induce ABCA1 expression and to polarize macrophages towards the M2 phenotype [51]. Consequently, 27-HC-treated macrophages are potent suppressors of T cell-functions. Both T cell proliferation and killing of target cells is reduced upon 27-HC treatment of macrophages. In conjunction, these effects lead to reduced anti-tumor activity of tumor-infiltrating T cells [51]. The clinical relevance of these in vitro observations is emphasized by the fact that the appearance of M2 macrophage infiltrates in epithelial ovarian cancer is associated with a poor prognosis [52]. Therefore, the identification of modulators of ABCA1 might be a novel strategy to prime the tumor environment towards tumor killing.

## 7. Conclusions and Perspectives

Although ABC transporters represent a large gene family in humans and mice and facilitate the transport of molecules important for many physiological functions and intercellular communication processes, our knowledge about these transporters in T cells is relatively sparse (Figure 3). This is in stark contrast to recent observations that T cells seem to be dependent on specific ABC transporters. In this context, murine effector Th17 (and Th1) cells were shown to be dependent on MDR1 and MRP4 in their pathogenic potential to mediate autoimmune intestinal inflammation [34,37]. In these studies, pharmacological blockade of these ABC transporters or of their specific substrates impaired intestinal inflammation in mice and a fraction of Crohn’s disease patients showed MDR1 loss of function [34,37]. Furthermore, a portion of human Th17 cells were reported to selectively express MDR1, which correlated with strong proinflammatory properties, including Th17 and Th1 cytokine production and a hypersensitivity to IL-23 stimulation [53].

The reason why we still know little about the role ABC transporters in T cell physiology might be due to the fact that ABC transporters represent a very heterogeneous group of transport proteins with a specificity for often several substrates per transporter, a matter which significantly complicates their experimental investigation. Although apparently selective pharmacological reagents for different ABC transporter exist, some studies recently observed off-target effects. Thus, investigating ABC transporters in T cells seem to necessitate thorough experiments using conditional genetic deletion approaches to investigate the function of ABC transporters. In addition, several substrates of ABC transporters are molecules that underly a complex synthesis and regulation in vivo, such as lipids or bile acid. This makes it, on the one hand, likely that T cells in specific environmental niches (consisting of a defined substrate composition) depend on distinct ABC transporters. However, this makes in vitro investigations relatively difficult as complex in vivo conditions cannot yet be sufficiently mimicked in in vitro culture systems, although strategies to overcome this are being discussed and established to mimic tumor microenvironments [46]. However, in vivo mouse models using genetically engineered mice with cell-type specific deletions of individual ABC transporters could expand our knowledge how ABC transporters regulate T cell-mediated physiological and detrimental immune responses. In this regard, data on Th2 cells in providing immunity to parasites or, on the flipside, in mediating allergic asthma or skin allergy are currently elusive, while data on Th17 cells already exist (Figure 3). Furthermore, it would be of significant interest to investigate whether the repertoire of ABC transporters used by Th2 and Th17 cells differs during physiologically and pathological immune responses, respectively. This is in fact not unlikely, as it is well accepted that the specific metabolic microenvironment in the same organ can change significantly when it is affected by a disease [54,55,56,57].

Future studies investigating the role of ABC transporters in T cells should also investigate whether and how deletion of distinct ABC transporters affects the metabolism of T cells. Many of the metabolites transported by ABC transporters were already shown to be important positive or negative regulators of different metabolic pathways utilized by T cells. The import of specific metabolites is not only a prerequisite for metabolic reprogramming and consecutive expansion of T cells, but also alters the production of fate-specific cytokines [55,58]. In addition, metabolic intermediates are known to contribute to ‘metabolic imprinting’ by epigenetic changes during chronic infections and/or posttranslational modification of proteins. In these molecular processes, ABC transporters are likely to play an important but so far neglected role.

Although the investigation of ABC transporters in T cells appears challenging, some groups have already shown that understanding how ABC transporters regulate T cell function can lead to the identification of novel drug targets to amplify immunity to viruses and tumors, or to mitigate autoimmunity or autoinflammatory disorders [29,30,34,37]. Especially the mobilization of memory T cell pools through pharmacological or genetic modulation of ABC transporters might be a promising therapy regimen for the treatment of various cancer subtypes [59,60] Vice versa, inhibition of ABC transporters, such as ABCG1, might proof suitable to promote the development of Tregs and, thereby, they could be helpful for treating chronic inflammatory and autoimmune diseases. In view of the pharmacological targeting of ABC transporters for these diseases, it must be taken into consideration that ABC transporters also fulfil essential physiological functions in several non-immunological tissues.
